# Anomalous SAXS at P12 beamline EMBL Hamburg: instrumentation and applications

**DOI:** 10.1107/S1600577521003404

**Published:** 2021-04-14

**Authors:** Andrey Yu. Gruzinov, Martin A. Schroer, Karen Manalastas-Cantos, Alexey G. Kikhney, Nelly R. Hajizadeh, Florian Schulz, Daniel Franke, Dmitri I. Svergun, Clement E. Blanchet

**Affiliations:** a European Molecular Biology Laboratory (EMBL), Hamburg Outstation c/o DESY, Notkestrasse 85, 22607 Hamburg, Germany; b Center for Data and Computing in Natural Science, University of Hamburg, Bundesstrasse 43, 20146 Hamburg, Germany; c Novartis, Novartis Campus, Fabrikstrasse 2, 4056 Basel, Switzerland; d Institute of Physical Chemistry, University of Hamburg, Grindelallee 117, 20146 Hamburg, Germany

**Keywords:** ASAXS, biological SAXS, metalloproteins, gold nanoparticles, anomalous scattering, beamline development

## Abstract

Small-angle X-ray scattering (SAXS) is an established method for studying nanostructured systems and in particular biological macromolecules in solution. To obtain element-specific information about the sample, anomalous SAXS (ASAXS) exploits changes of the scattering properties of selected atoms when the energy of the incident X-rays is close to the binding energy of their electrons. While ASAXS is widely applied to condensed matter and inorganic systems, its use for biological macromolecules is challenging because of the weak anomalous effect. Biological objects are often only available in small quantities and are prone to radiation damage, which makes biological ASAXS measurements very challenging. The BioSAXS beamline P12 operated by the European Molecular Biology Laboratory (EMBL) at the PETRA III storage ring (DESY, Hamburg) is dedicated to studies of weakly scattering objects. Here, recent developments at P12 allowing for ASAXS measurements are presented. The beamline control, data acquisition and data reduction pipeline of the beamline were adapted to conduct ASAXS experiments. Modelling tools were developed to compute ASAXS patterns from atomic models, which can be used to analyze the data and to help designing appropriate data collection strategies. These developments are illustrated with ASAXS experiments on different model systems performed at the P12 beamline.

## Abstract 2

Hardware and software for anomalous small-angle X-ray scattering on biological macromolecules in solution implemented at the P12 beamline of the EMBL (PETRA III storage ring, DESY, Hamburg) are described.

## 1. Introduction   

Small-angle X-ray scattering (SAXS) is a powerful method to study macromolecular solutions. SAXS is commonly used to probe the folding state, interactions and flexibility of proteins, structures of macromolecular complexes, and the structural responses to variation in external conditions, yielding low resolution information on the size and shape of the particles (Svergun *et al.*, 2013 ▸; Schroer & Svergun, 2018 ▸). In a SAXS experiment, the sample is illuminated by a monochromatic X-ray beam and the scattered X-ray photons are collected as a function of the scattering angle as a two-dimensional image. Such two-dimensional images are typically isotropic and azimuthally averaged to obtain the intensity *I* versus *s* = 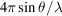, where λ is the wavelength of the incoming radiation of energy *E* and 2θ is the scattering angle. The resulting one-dimensional scattering pattern can then be analyzed to extract structural information on the sample at the nanometre scale.

At a fundamental level, the interaction of X-rays with the sample effects in Thomson scattering from electrons. The electrons excited by the incoming X-rays become a secondary source of electromagnetic waves and emit photons with the same energy as the incident ones. The radiation emitted by these secondary sources in turn interfere in a constructive way and an interference pattern is recorded that contains information about the electron density distribution in the sample. For a particle with known atomic structure one can calculate the isotropic SAXS intensity using the Debye formula (Debye, 1915 ▸),
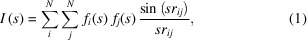
where *N* is the number of atoms within the particle, *f*_*i*_ and *f*_*j*_ are the scattering amplitudes from the *i*th and *j*th atom, respectively, and *r*_*ij*_ is the Euclidian distance between these two atoms.

**Figure 1:**
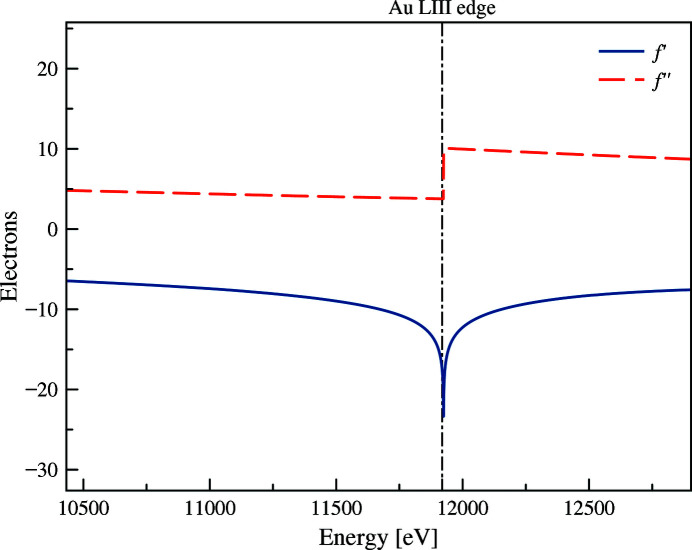
The energy-dependent real and imaginary parts (*f*′ and *f*′′, respectively) of the X-ray scattering factor of gold near its *L*_III_-edge of 11919 eV (vertical dash-dotted line), depicting the anomalous effect. Data are retrieved from http://skuld.bmsc.washington.edu/scatter/AS_periodic.html.

When the energy of the incoming X-rays is close to the binding energy of a core electron in an atom, the electron can be ejected, and an outer shell electron can fill the vacancy within the inner shell, emitting a fluorescence X-ray photon. As a consequence, the scattering amplitude of the resonant atom changes at the X-ray absorption edge leading to changes of the resulting scattering pattern of the entire object. Anomalous SAXS (ASAXS) exploits these element-specific changes to gain information on the distribution of the resonant atoms within the sample. The X-ray scattering factor of an atom as a function of photon energy *E*, *f*(*s*, *E*), is expressed as
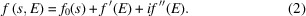
Here, *f*_0_(*s*) is the atomic scattering factor far from the absorption edge and *f*′ and *f*′′ are the energy-specific correction terms, which become significant contributors to the atomic scattering amplitude if *E* is close to an X-ray absorption edge of the atom (Fig. 1 ▸). These corrections are calculated from absorption or fluorescence measurements for a particular sample (Evans & Pettifer, 2001 ▸). If this is not possible, theoretical values for different atoms can be found in the literature (Cromer & Liberman, 1981 ▸; Cromer & Mann, 1968 ▸).

By collecting several SAXS patterns at different X-ray energies (wavelengths) close to the absorption edge, the differences in the scattering patterns arising from variations in the scattering factor of the resonant atom can be detected. Analysis of these differences provides information on the distribution of atoms of interest. Anomalous scattering is widely exploited in macromolecular crystallography for experimental phase determination (Hendrickson & Ogata, 1997 ▸), where the signal is amplified due to the repeating intermolecular distances within the crystal. Less frequent are ASAXS experiments on biological molecules due to the usually rather weak anomalous effect.

Instead, ASAXS is employed for studying materials with high scattering contrast, such as alloys or glasses (Hoell *et al.*, 2014 ▸; Tuaev *et al.*, 2013 ▸; Tatchev *et al.*, 2011 ▸). In particular, ASAXS was used to determine the nanostructure, spatial arrangement and the concentration of calcium in nanoparticles embedded in a silicate glass matrix (Hoell *et al.*, 2014 ▸). The number of publications mentioning ‘ASAXS’ are shown in Fig. S1 of the supporting information.

Pioneering ASAXS experiments on weakly scattering samples such as soft matter and biological macromolecules in solution at very high concentration were performed in the 1980s (Stuhrmann, 1980 ▸, 1981*a* ▸,*b*; Miake-Lye *et al.*, 1983 ▸). However, ASAXS on biological samples remains challenging due to multiple reasons: (i) these samples are largely composed of lightweight atoms without absorption edges in the hard X-ray regime, (ii) the SAXS signal is usually rather weak, the ASAXS signal even weaker, (iii) samples are often limited in quantity and (iv) are sensitive to radiation damage (Jeffries *et al.*, 2015 ▸). These experimental limitations can explain the relatively few examples of biological ASAXS studies published over the last decades, while standard biological SAXS on macromolecules has been attracting a growing number of users (Schroer & Svergun, 2018 ▸).

Despite these challenges, ASAXS can be successfully applied to study systems with sufficient numbers of anomalous atoms. It was used, for example, to describe the counter-ion distribution around DNA and other biopolymers in solutions such as polyaspartic acid, chondroitin sulfate, hyaluronic acid (Pabit *et al.*, 2010 ▸; Horkay *et al.*, 2018 ▸) and micelles (Sztucki *et al.*, 2011 ▸, 2012 ▸; Jusufi *et al.*, 2012 ▸). ASAXS was also employed to characterize the core of micelles formed by bromine-containing amphiphilic block copolymers (Akiba *et al.*, 2012 ▸). The ASAXS approach was used on platinum-containing hydro­gels, where the contribution of the different components could be separated, and the size distribution of the platinum particles as well as the internal structure of the polymer complexes were obtained (Svergun *et al.*, 2001 ▸). Other examples of biological ASAXS include the determination of the terbium ion distribution in centrifugally oriented acetyl­choline receptor-enriched membranes (Lee *et al.*, 2009 ▸) and the characterization of the ionic environment surrounding protein–spherical nucleic acid conjugates used in DNA-driven crystal engineering strategies (Krishnamoorthy *et al.*, 2018 ▸). To utilize the anomalous scattering effect, nanoparticle labelling can be employed to determine intramolecular distances with relatively good precision without significantly altering the structure of the molecule under investigation (Zettl *et al.*, 2016 ▸).

Several examples of distance determination within a biological macromolecule using only a few resonant atoms have been reported (Stuhrmann & Notbohm, 1981 ▸; Meisburger *et al.*, 2015 ▸; Makowski, 2010 ▸). However, to cope with the weak anomalous signal protein concentrations up to 350 mg ml^−1^ were utilized, two orders of magnitude higher than those employed in standard biological SAXS measurements.

ASAXS is often performed around the absorption edges of metals due to the accessibility of these energies on the modern synchrotron sources and can be interesting for the study of large metalloproteins complexes. Metals play an important role in biochemistry, in particular in catalysis. *In vitro* metal ions bind to the protein sites based on the Irving–Williams series, suggesting that the metal–protein complexes’ stability decreases, depending on which ion is introduced (Irving & Williams, 1953 ▸). The relative abundance of the inorganic elements generally present in enzymes is depicted in Fig. S2 of the supporting information. Here, magnesium is the most abundant metal but its absorption edge of 1.3 keV is below the available range on most of the SAXS beamlines. Moreover, this would require extremely thin sample capillary wall thicknesses to reduce the X-ray absorption to an acceptable level.

Another application of metals in biochemistry is, for example, the usage of large molecules such as apoferritin as containers for metal binding, uptake and further delivery into the cell. Apoferritin can store about 4500 iron atoms in Fe^3+^ state as a mineral. Giving its storage properties, ferritin is utilized as potential drug nanocarrier (Wang *et al.*, 2019 ▸) and as a nanocage for growing small monodisperse nanoparticles (Kasyutich *et al.*, 2010 ▸). Such nanocontainers can be produced in large quantities and have a high number of metal atoms inside, making them suitable for ASAXS to gain insights into these medically important objects.

To measure the unique structural information provided by ASAXS on biologically relevant solutions, including also pharmaceutical molecules, the aforementioned limitations of this technique need to be adequately addressed. Here, we describe the effort to develop a reliable ASAXS environment at the P12 BioSAXS beamline. This work capitalizes on the energy tunability of the beamline and its merits for the data collection of biological samples including low instrumental background, beam stability, automated sample handling and data processing. Procedures to reliably collect scattering data at different energies close to the absorption edge were developed together with data analysis algorithms allowing to correct for X-ray fluorescence and to extract the anomalous signal. A number of tools were developed for (i) computation of the ASAXS signal from atomic models (existing or expected), (ii) facilitating the design of the actual experiment and (iii) data analysis. The full workflow to perform ASAXS experiments at P12 is described here. Its application is further illustrated with the experiments and analysis on several test samples containing different anomalously scattering atoms.

## 2. The ASAXS experiment   

### 2.1. SAXS data collection at the P12 beamline   

The P12 beamline operated by the EMBL and located at the PETRA III storage ring (DESY, Hamburg, Germany) is dedicated to, and optimized for, biological SAXS. The instrumental background is reduced by using scatterless slits and an in-vacuum flow-through capillary, allowing to collect the weak scattering signal of proteins in solution (Blanchet, Spilotros *et al.*, 2015 ▸). Scattered photons are collected on a hybrid photon-counting Pilatus 6M detector (DECTRIS, Villigen, Switzerland) (Broennimann *et al.*, 2006 ▸). Different sample-to-detector distances (from 1.6 to 6 m) can be used to cover different ranges of scattering angles. The transmitted beam intensity is measured by a diode mounted in the beamstop (Blanchet, Hermes *et al.*, 2015 ▸) and used to normalize the scattering pattern.

An automatic sample changer (Round *et al.*, 2015 ▸) allows sample loading to the measurement cell, flow the sample in the cell during exposure, and clean and dry the cell in between measurements (Graewert *et al.*, 2015 ▸; Hajizadeh *et al.*, 2018 ▸). The SAXS data analysis pipeline *SASFLOW* generates a table with the main SAXS-derived structural parameters such as overall shape and characteristic size, *i.e.* radius of gyration *R*_g_ and maximal shape size *D*_max_, volume and molecular weight, as well as three-dimensional low resolution bead models within minutes after data collection (Franke *et al.*, 2012 ▸).

Samples are generally measured in between two corresponding buffers such that the background scattering can be accurately determined and subtracted from the scattering of the sample to isolate the contribution of the pure solute. Scattering from the empty capillary and pure water is collected for absolute calibration of the beamline intensity, or, alternatively, a protein sample of known molecular weight and concentration is collected for the relative calibration. In addition, for ASAXS data collection, an additional sample is typically measured which does not contain the anomalously scattering atoms. This way the impact of a possible change in the beamline background after energy adjustment can be evaluated and ensured that no bias is introduced by these changes for the ASAXS data analysis.

### 2.2. Energy adjustment   

Anomalous SAXS involves repetitive measurements at different energies close to the absorption edge of the resonant atom. The scattering amplitude *f*(*s*, *E*) of the resonant atoms varies mostly within a few eV at the absorption edge and ASAXS requires accurate selection of the incoming X-ray energy. P12 is equipped with a double-crystal monochromator (DCM) with Si(111) crystals, which allows for energy selections between 4000 and 20000 eV (λ = 0.06–0.3 nm) (Blanchet, Spilotros *et al.*, 2015 ▸). Si(111) crystals offer energy resolution (Δλ/λ) of 10^−4^, which is sufficient for typical ASAXS measurements (Sztucki *et al.*, 2010 ▸).

The calibration of the monochromator and the adjustment of the energy are implemented in a set of *Python* scripts accessible through the beamline graphical user interface (Hajizadeh *et al.*, 2018 ▸) (see details in Section S3).

A list of chemical elements with absorption edges between 3500 and 20000 eV is presented in Table 1 of Section S4 of the supporting information. Absorption edges in the range 6000–20000 eV can be utilized at the P12 beamline. The lower part of the monochromator energy spectrum is not fully accessible due to absorption by a safety vacuum window and the sample cell.

### 2.3. Experimental parameters   

#### 2.3.1. Data collection   

Two alternative approaches can be employed for ASAXS data collection. In the first approach, the sample flows through the cell while the different beam energies are set and multiple SAXS patterns are collected. This mode of measurement is rather fast, and allows to collect SAXS patterns at 20 different energies within 3 min. This approach is, however, limited to radiation-resistant samples or may be utilized for fast initial assessments.

For radiation-sensitive solutes another method of measurement is needed, where fresh samples are loaded for each energy. In practice, the beamline is set at the first energy, samples, buffer, and potential additional standard sample are measured, then this procedure is repeated for each energy. The best data collection procedure is chosen on a case-to-case basis. The samples can be measured first in the fast mode with 15–20 energy points to help select the most suitable energies, that will be used for the second mode of data collection.

In the present study, all samples were measured in a flow-through capillary of 0.9 mm diameter (Schroer *et al.*, 2018 ▸) using a robotic sample changer and a sample-to-detector distance of 3.1 m. The intensity of the incoming beam has been estimated by measuring the scattering from a thin foil positioned on the beam path upstream of the sample cell and the intensity of the transmitted beam by the active beamstop (Blanchet, Hermes *et al.*, 2015 ▸). These two intensities are later used to obtain the absorption spectra.

The fluorescence signal at and above the edge is isotropic and results in a background increase in the SAXS image. By estimating the background change in the SAXS data, fluorescence spectra can be determined (see automatic fluorescence constant detection described in Section S5 of the supporting information). Absorption or fluorescence spectra can be used for the calculation of experimental values of anomalous corrections *f*′ and *f*′′ using the program *CHOOCH* (Evans & Pettifer, 2001 ▸).

To calibrate the collected data frames, empty capillary, water and protein solutions without anomalous atoms (using for example bovine serum albumin or lysozyme) are measured at each energy. Specifically developed ASAXS Python scripts running within the *BECQUEREL* beamline control software (Hajizadeh *et al.*, 2018 ▸) allow the beamline users to queue measurements of the samples and standards for all energies so that the data collection can run in a fully automated mode.

#### 2.3.2. Sample preparation   

Tetra­decyl­tri­methyl­ammonium bromide (TTAB) powder was obtained from Sigma-Aldrich (product number T4762). Solutions were prepared at concentrations above the TTAB critical micelle concentration (c.m.c.) of 50 m*M*, using MilliQ water (Millipore, Massachusetts, USA). Energies near the bromine *K*-edge (13474 eV) were used. Measurements were made in the fast mode, *i.e.* using continuous flow of the solution in the capillary with simultaneous change of energy during data collection.

Nanoparticles with 10 nm average diameter gold core, covered with silica, were obtained from Sigma-Aldrich and used as is (product number 747564, with reported 9–12 nm core diameter and 18–22 nm silica shell thickness). In addition, gold nanoparticles of radius *R* = 4.1 nm with a low size polydispersity (Δ*R*/*R* = 11%) coated with α-meth­oxy­poly(ethyl­ene glycol)-ω-(11-mercaptoundecano­ate) (PEGMUA) ligands of two molecular weights (2 kDa, 5 kDa) were synthesized according to the procedure described by Schulz *et al.* (2013 ▸, 2016 ▸). Concentration series made from both types of sample systems show no aggregation. The gold nanoparticle solutions were measured at energies close to the *L*_III_ absorption edge of gold (11919 eV).

### 2.4. ASAXS data reduction and analysis   

#### 2.4.1. Initial data reduction   

The calibration of the angular axis was made by the powder diffraction pattern of silver behenate measured at a defined energy (for example at the edge of the element of interest). For each energy, the angular scale was appropriately adjusted to take into account the change in the wavelength, and the recorded intensities of the SAXS signal were scaled according to the procedure described by Zettl *et al.* (2016 ▸). Initial data reduction including the *s*-range adjustment and normalization to the transmitted beam for absolute intensity at each energy was incorporated into the *SASFLOW* pipeline used routinely at the P12 beamline (Franke *et al.*, 2012 ▸).

#### 2.4.2. Fluorescence background correction   

In conventional SAXS, the scattering form factor of the solute is obtained by subtracting the SAXS pattern collected on the buffer from the data collected on the sample, thus subtracting the contribution from the buffer surrounding the solute and also the instrument background, *e.g.* the scattering from the measurement cell. In the case of anomalous SAXS, at the energies close to and above the absorption edge, fluorescence photons are produced resulting in a constant offset in the SAXS curves. The fluorescence contribution can be difficult to evaluate and sometimes impossible to accurately subtract as it comes from the anomalously scattering atoms in the sample, which are not necessarily present in the buffer. Reliable fluorescence correction can be made by using a fluorescence detector built into the measurement setup. Data analysis should be processed only after fluorescence correction of the initial data.

**Figure 2:**
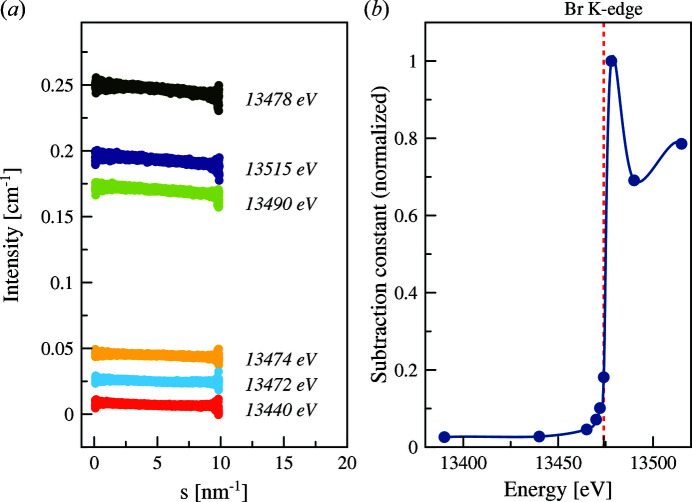
(*a*) SAXS scattering curves from potassium bromide solution in water measured at different energies. Measurements close to the *K*-absorption edge of bromine (13474 eV) produce an increase in the constant background mainly due to fluorescence. (*b*) Normalized constant offset determined for each curve in (*a*) plotted against energy of incoming X-rays. Intermediate energy points are also shown. The dashed line shows the position of the bromine *K*-edge.

Fluorescence contributes to the integrated scattering curves as the constant uniform energy-dependent background that usually manifests in ‘undersubtraction’ in resulting buffer-subtracted scattering curves [Fig. 2 ▸(*a*)]. Due to the intrinsic resolution limit of the monochromator (typically several eV), a constant term correction, which includes mainly fluorescence and Raman scattering, has to be taken into account when considering the data not only above the absorption edge but already starting from energies slightly below the absorption edge. Generally speaking, such a constant contribution accounts for the angle-independent fluctuation scattering, resonant Raman scattering and fluorescence effects.

Optional automatic estimation of the constant energy-dependent shift of the scattering intensity in buffer-subtracted scattering curves due to the onset of the fluorescence was implemented in the data processing pipeline for a rapid assessment of the data quality of the data.

As can be seen in Fig. 2 ▸(*b*), the constant term is close to zero at some energies (*e.g.* at 13440 eV). In the vicinity of the abortion edge [dashed red line in Fig. 2 ▸(*b*)] the constant is much larger mainly due to the onset of fluorescence. Such an approximate estimate of fluorescence contribution should be used with caution; however, it allows one to quickly assess the position of the absorption edge of the element under investigation solely from the buffer-subtracted scattering curves. Details of the automatic fluorescence constant background estimation can be found in Section S5.

### 2.5. ASAXS data analysis   

#### 2.5.1. ASAXS decomposition   

The SAXS intensity at energy *E* over the range of scatting vector *s* reads as (Stuhrmann, 1980 ▸, 1981*b* ▸)
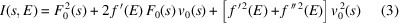
where 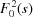 is the non-resonant scattering intensity far from the absorption edge, 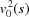 the resonant scattering intensity from the spatial distribution of anomalous atoms, and *F*_0_(*s*)*v*_0_(*s*) is the cross-term represented as a product of non-resonant and resonant scattering amplitudes. While 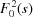 corresponds to the Fourier transform of scattering length density, and thus the electron density contribution from the entire particle, 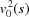 is related solely to the anomalous scattering length density and reflects the distribution of the anomalously scattering atoms. For ASAXS experiments, SAXS curves are collected at different energies close to the respective absorption edge where the variation of *f*′ and *f*′′ are large, such that the non-resonant, resonant and cross term intensity can be determined.

If at least three energy measurements are performed, the system of linear equations (3) can be solved (Sztucki *et al.*, 2012 ▸) to extract the resonant scattering intensity term 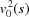. Further energy points can be measured to provide more stable results of this decomposition. A recent approach was developed to analyze ASAXS from two-phase alloys which discusses more thoroughly the problem of ill-posed systems of linear equations (Goerigk, 2018 ▸).

The contribution *f*′ has a jump at the edge, while *f*′′ before and after the absorption edge changes weakly and monotonically with the energy (*cf*. Fig. 1 ▸). Therefore, another approach is to neglect *f*′′ in equation (3) and use only the energies below the absorption edge. A quadratic approximation for the dependence of *I*(*E*, *f*′) at different energies and fixed *s* yields to
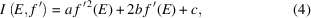
where the coefficients are equal to the corresponding scattering parts of decomposition: *a* = 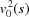, *b* = *F*_0_(*s*)*v*_0_(*s*), *c* = 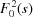 (Ballauff & Jusufi, 2006 ▸).

The matrix decomposition method (3) has the advantage of using all measured energies whereas the parabola method (4) only exploits energies below the absorption edge. Additionally, equation (4) is only usable near and below *K*-edges. Only in those cases is *f*′′ close to zero. At *L*_III_ edges, *f*′′ cannot be neglected.

#### 2.5.2. Impact of the anomalous scattering on the apparent radius of gyration   

The single particle intensity after spherical averaging can be expressed using the Guinier approximation for small *s* as
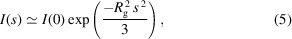
where *R*_g_ is the radius of gyration of the particle and *I*(0) is the forward scattering. *R*_g_ depends on the electron density distribution within the particle – it is related to the shape and provides an indication of the particle compactness. *R*_g_ can also be assessed from the particle distance distribution function *p*(*r*) as
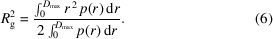
The function *p*(*r*) is the distribution of distances between volume elements inside the particle weighted by their excess scattering densities and it is related to the scattering intensity *I*(*s*) by an inverse Fourier transform,
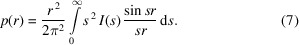
Generally, *R*_g_ of a solid sphere is smaller than that of a spherical shell with the same outer radius because of a reduced contribution of smaller intraparticle distances in the latter case. In the case of anomalous scattering, the scattering length density of the anomalous atoms decreases at their absorption edges, thus decreasing the contribution of these atoms to the scattering pattern and to the corresponding distance distribution. When the anomalous atoms are distributed mostly at the periphery of the particle, their distances from the origin are on average larger. At the edge, with the decrease of contribution from these larger distances, the apparent *R*_g_ decreases. When the anomalous atoms are distributed in the core, then the relative contribution of smaller distances will be smaller and the apparent radius of gyration will increase. Therefore, plotting the computed *R*_g_ versus the X-ray energy yields an immediate information on the overall distribution of the anomalous atoms within the particle.

## 3. ASAXS curves computation   

### 3.1. Core-shell spherical model   

The solution SAXS profiles of macromolecules with known atomic structure can be computed and used to fit to experimental data using the program *CRYSOL* from the *ATSAS* package (Svergun *et al.*, 1995 ▸). *CRYSOL* utilizes spherical harmonics, which significantly reduces the calculation time compared with the Debye formula.

In the anomalous scattering mode of *CRYSOL*, anomalous correction factors *f*′ and *f*′′ that were calculated using the approach described by Cromer & Liberman (1970 ▸, 1981 ▸) are added to the absorbing atom’s X-ray scattering form factor as described by equation (2). For more details of the method implementation, refer to Manalastas-Cantos *et al.* (2021 ▸).

Computation of anomalous scattering curves from atomic structure and the decomposition procedures were first tested using a simple model. Scattering curves were computed from artificially constructed spheres with gold core and silica shell. These spheres were generated using the *packmol* software based on the concept of packing optimization such that each type molecule must satisfy spatial constraints and the distance between atoms of different molecules must be greater than some specified tolerance (Martínez *et al.*, 2009 ▸).

The sphere was modelled as a gold core with a radius of 5 nm and a 37 nm-thick silica shell. Based on the allocated volume, 36928 gold atoms (hexagonal packing) were used for the core and 4665627 silica molecules for the shell with a total number of 1.4 × 10^7^ atoms. The model PDB file was used by *CRYSOL* to compute the scattering curves at the energies of 11000, 11500, 11700, 11800, 11850, 11900, 11919 (edge), 11925, 11950, 12050 and 12100 eV.

**Figure 3:**
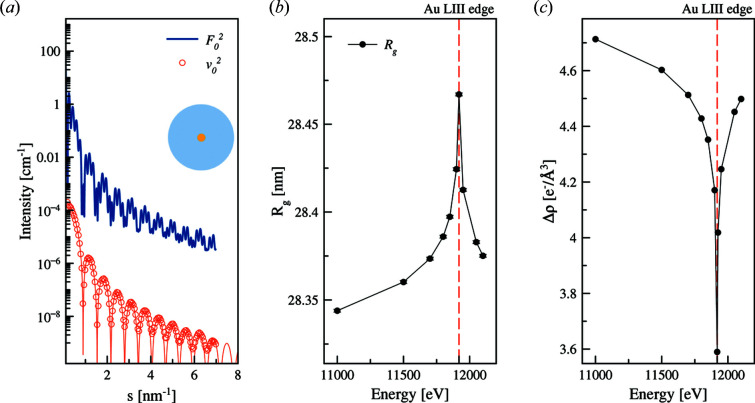
(*a*) Scattering from a model containing a gold core and silica shell (inset) calculated using *CRYSOL* assuming the sample concentration of 1 mg ml^−1^. Blue solid line: scattering far from the gold absorption edge; red circles depict the extracted anomalous contribution. Oscillations are present due to strong scattering contrast between the gold core and silica shell. The solid red line represents the theoretical scattering from a sphere with a radius *r* = 5 nm that coincides with extracted scattering from a gold core of the same size. (*b*) Radius of gyration computed for a set of modelled curves around the absorption edge of gold (11919 eV). The peak value corresponds to the edge position (dashed line). Errors are smaller than the symbol size. (*c*) Contrast of the gold core at different energies.

Energies below the absorption edge were selected to estimate the anomalous contribution from the matrix decomposition method. The resulting scattering curves and the variation are shown in Fig. 3 ▸(*a*). The gyration radii *R*_g_ around the absorption edge of gold are shown in Fig. 3 ▸(*b*). The resonant scattering intensity (anomalous term) reflects the scattering from a sphere with radius of 5 nm which is in perfect agreement with the core size of the model.

*R*_g_ appears to have a maximum at the absorption edge (Δ*R*_g_ = *R*_edge_ − *R*_far_ = 0.123 nm). This is explained by the decrease of the small distances’ contribution by anomalous gold at the absorption edge. Contrast of the gold core decreases in the vicinity of the absorption edge [Fig. 3 ▸(*c*)]. The increase of the apparent *R*_g_ in the vicinity of the absorption edge reveals that the anomalous atoms are distributed inside the gold core as described in Section 2.5.2.

### 3.2. Computation of ASAXS curves from atomic structures   

ASAXS on biological macromolecules is notoriously difficult due to the weak anomalous signal and possible radiation damage. To have a better idea of the applicability of ASAXS, theoretical anomalous effects have been computed from high-resolution models from the Protein Data Bank (PDB) (https://www.rcsb.org/). A screening was first conducted to identify entries containing atoms whose absorption edges can be accessible for X-ray experiments (*e.g.* iron, cobalt, zinc, gold *etc*.). These entries were selected using the freely available *pypdb* toolkit (Gilpin, 2015 ▸). About 30% of all PDB entries contain elements whose absorption edge can be accessed on the P12 beamline (a full summary is available in Table 2 in Section S6).

In order to estimate the anomalous scattering effects, we have screened the PDB for the number of metal ions and used an empiric approach. For each metal ion, we used *CRYSOL* to compute SAXS curves with and without anomalous corrections. The computed curve with the maximum anomalous effect (*i.e.* at the absorption edge) was divided by the one without the effect to determine the percentage of the ASAXS signal at the absorption edge as a function of *s*.

**Figure 4:**
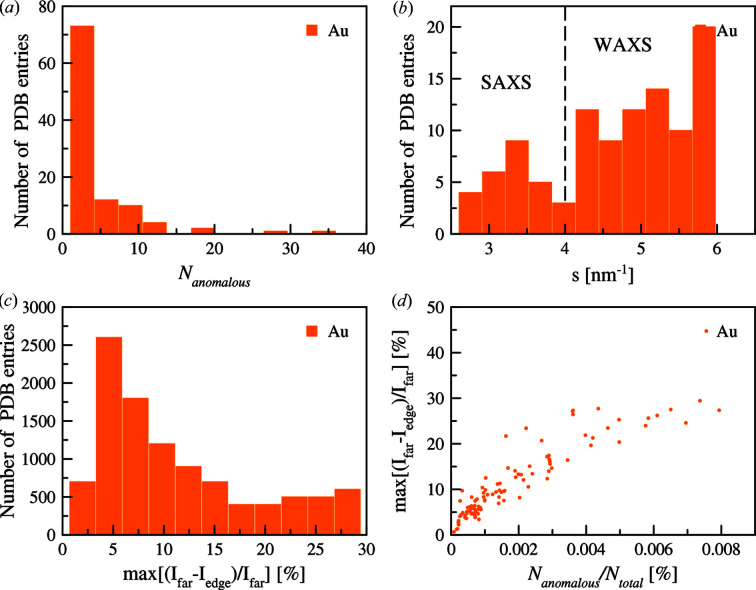
Statistics on the PDB entries containing gold atoms. (*a*) Distribution of the number of gold atoms in the structures. (*b*) Distribution of the *s*-range where a maximum of the intensity difference is located. (*c*) Distribution of the ratio of maximum intensity difference due to the anomalous effect. (*d*) Ratio of anomalous atoms in the structure as a function of the computed ratio of the maximum intensity difference.

Gold atoms were used for a first example. Due to the limited number of entries (107) this example allows for a rapid computation and overview of the statistical results (Fig. 4 ▸). For each protein, the angular position where the difference between energies at and off the resonance is maximal was determined.

Fig. 4 ▸(*a*) shows the distribution of the number of gold atoms in gold-containing PDB structures. The majority of gold-containing high-resolution structures contain less than ten gold atoms. After computing the ratio of the scattering curves at and far from the resonance energy, the intensity variation and the position of the maximum variation can be determined [Figs. 4 ▸(*b*) and 4(*c*)]. The difference was distributed in two parts: one is up to 4 nm^−1^, *i.e.* within a typical range of SAXS measurements, and the wide-angle part [Fig. 4 ▸(*b*)]. The contribution of the anomalous atoms produces the average percentage of intensity difference of around 10% [Fig. 4 ▸(*c*)]. This emphasizes the need of reliable intensity correction and low background of the instrument to obtain measurable differences in the scattering curves. Fig. 4 ▸(*d*) demonstrates that the maximum intensity difference versus percentage of the anomalous atoms increases linearly in the beginning, but tends to show saturation for larger numbers of gold atoms in the structure.

**Figure 5:**
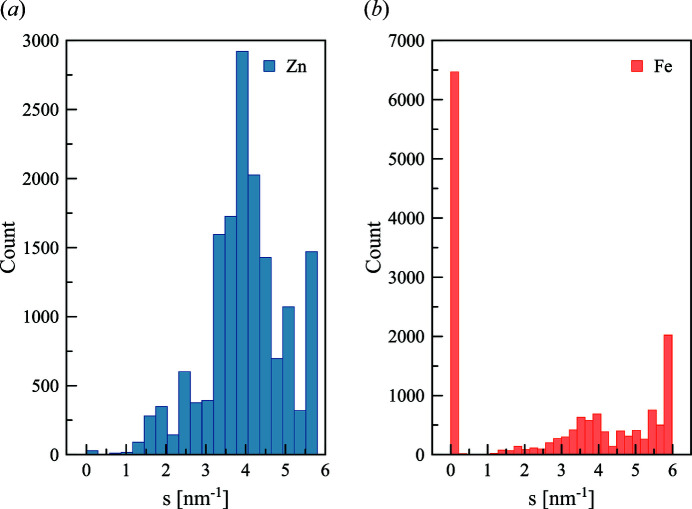
Distribution of the angular range for maximum intensity difference due to the anomalous effect for the PDB entries containing zinc and iron atoms.

The same approach was used with zinc- and iron-containing PDB files (16125 and 9226 entries, respectively), that are among the most abundant atoms with the edges accessible at the P12 beamline. The zinc- and iron-containing structures show the same trend as for gold atoms (Fig. 5 ▸). Interestingly, the main difference in the scattering intensity is located between 2 and 5 nm^−1^ for both cases, which is a typical SAXS range measured on many SAXS instruments. Most of the iron-containing structures displayed the largest differences at the very small angles (up to 0.5 nm^−1^).

**Figure 6:**
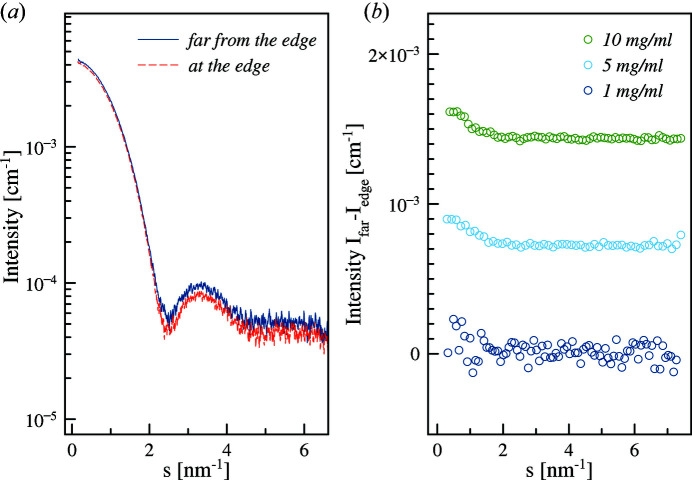
(*a*) Computed curves far from the absorption edge and at the absorption edge from parvalbumin (PDB: 4cpv) with calcium atoms substituted by terbium (absorption edge 7514 eV) at a protein concentration of 10 mg ml^−1^. (*b*) Difference in the intensity between the computed curves far from the absorption edge and at the absorption edge from parvalbumin (PDB: 4cpv) with calcium atoms substituted by terbium (absorption edge 7514 eV) at different solute concentrations. The curves are shifted for the better representation.

In order to estimate the sample concentration required to detect the anomalous signals, data were simulated using *CRYSOL* and *IMSIM* (Franke *et al.*, 2020 ▸). Fig. 6 ▸ displays theoretical ASAXS patterns computed from calcium-liganded parvalbumin (Miake-Lye *et al.*, 1983 ▸) (PDB: 4cpv) where terbium atoms (absorption edge 7514 eV) were introduced in place of two calcium atoms (two atoms with about 1.2 nm separation in the structure). The difference curves were obtained by subtracting the scattering signal at the absorption edge from one far from the absorption edge. The scattering signal from proteins at different concentrations as well as the buffer signal was modelled using program *IMSIM*. One can see that the actual difference in scattering intensity stays rather small even for a protein concentration of 10 mg ml^−1^.

Based on an absolute intensity estimation from a typical photon flux of 5 × 10^12^ photons s^−1^ and 1 s exposure time for a parvalbumin solution with molecular weight of 11.8 kDa enriched with two terbium atoms, one needs to have an approximately 10 mg ml^−1^ solution of protein to be able to reliably distinguish anomalous signal in real experimental settings. In the original study (Miake-Lye *et al.*, 1983 ▸) the estimated concentration of the protein of approximately 350 mg ml^−1^ was used.

The utilized combination of *CRYSOL* and *IMSIM* allows for a rapid assessment of the expected anomalous effect from macromolecular solution. This procedure may be helpful to optimize the experimental parameters for data collection such as exposure time, sample concentration and angular range.

## 4. Experimental data   

### 4.1. Tetra­decyl­tri­methyl­ammonium bromide (TTAB)   

**Figure 7:**
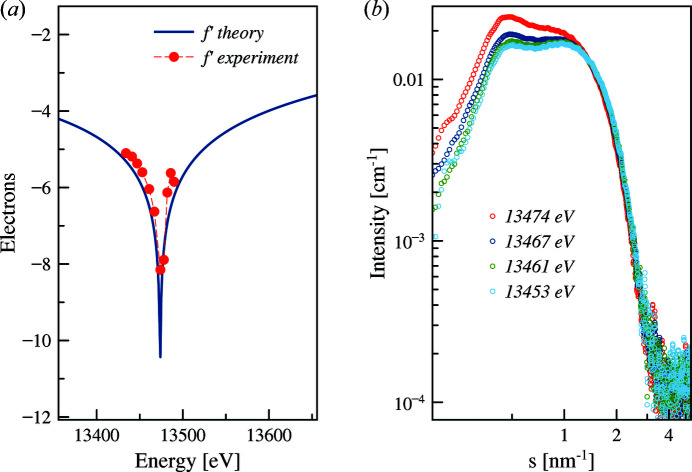
(*a*) Theoretical (solid line) and experimental (red dots) *f*′ correction in the vicinity of the absorption edge of bromine. Theoretical values are retrieved from http://skuld.bmsc.washington.edu/scatter/AS_periodic.html. (*b*) ASAXS from TTAB micelles across the bromine edge (13474 eV). Changes in the beginning of the curve reflect the changes of scattering factors of bromine ions surrounding the micelle in solution

Tetra­decyl­tri­methyl­ammonium bromide (TTAB) is a cationic surfactant, which assembles into micelles. The positive charges of the tri­methyl­ammonium heads are screened by bromine ions. Conducting ASAXS at the bromine edge allows one to study the counter ion distribution around the micelles. This system has been measured on other SAXS instruments (Jusufi *et al.*, 2012 ▸; Sztucki *et al.*, 2011 ▸) and is used here as a standard to evaluate the possibilities of the P12 beamline. The measurements were made using simultaneous energy changes and sample flow through the capillary with an overall sample–buffer measurement time of 3 min in total. An X-ray absorption spectrum was used to determine the anomalous factors for this sample (Fig. 7 ▸).

The measured ASAXS curves were decomposed into energy-independent SAXS terms using equation (3). The resulting anomalous signals were averaged to increase the signal-to-noise ratio. Although it is reported that 50 m*M* TTAB has a significant structure factor, the anomalous effect in the structure factor is weak compared with that in the form factor (Sztucki *et al.*, 2012 ▸). High concentrated samples of 50 m*M* (17 mg ml^−1^) were used to obtain better statistics for the decomposition procedure.

**Figure 8:**
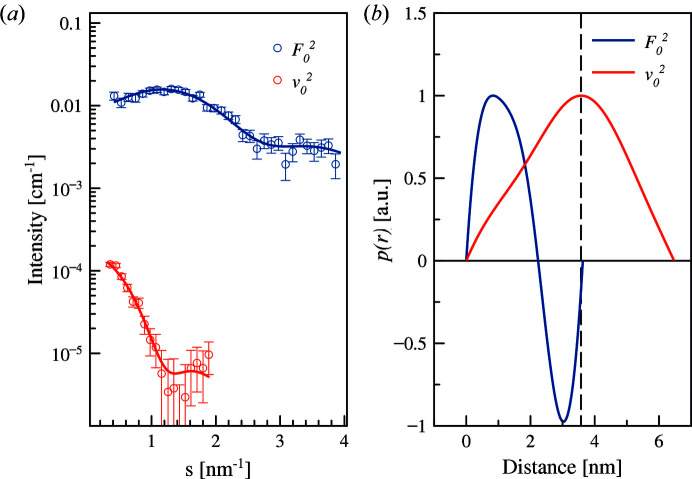
(*a*) Conventional (blue) and anomalous (red) scattering SAXS curves from TTAB-Br micelles. Solid lines represent the fits by the *GNOM* software (Svergun, 1992 ▸). The number of shown experimental points is reduced for clarity. (*b*) Resulting normalized pair distance distribution function *p*(*r*) as described in equation (7) computed based on the anomalous (red) and conventional (blue) components. The dashed line shows the position of the maximum of the anomalous function.

The anomalous and non-anomalous components of the TTAB-Br micelles scattering computed using the matrix decomposition method are shown in Fig. 8 ▸(*a*); the pair distance distribution function of the anomalous component is typical for a hollow particle displaying a broad peak around 3.6 nm and with maximum size of 6.2 nm. These values are in good agreement with the radius of the shell, 3.1 nm, determined by Sztucki *et al.* (2010 ▸) based on a direct fitting of the data with the form factor of a hollow sphere. The pair distance distribution function of the non-anomalous component is typical for a particle with different contrasts with respect to the outer solution; hydro­philic heads and hydro­phobic tails of amphiphilic molecules are self-organizing to micelles (Svergun *et al.*, 1995 ▸).

For this sample the scattering density of the core (formed by the surfactant) is lower than that of the solvent (water), while the scattering density of the bromine atom moiety is higher than that of water. As a consequence, the measured SAXS curves have a characteristic downturn at smaller scattering angles (which normally points to repulsive interparticle structure factors but in this particular case it is caused by the particular scattering length distribution within the particle). Therefore, for this system, a straightforward use of the Guinier approximation would not have been possible.

Overall, TTAB micelles show a well pronounced anomalous scattering effect corresponding to the bromine ion cloud surrounding the particles. The anomalous scattering contribution can be reliably extracted from the series of measurements at different energies and reveals a specific condensation of counter-ions onto the micelle surface.

### 4.2. Gold nanoparticles coated with silica   

**Figure 9:**
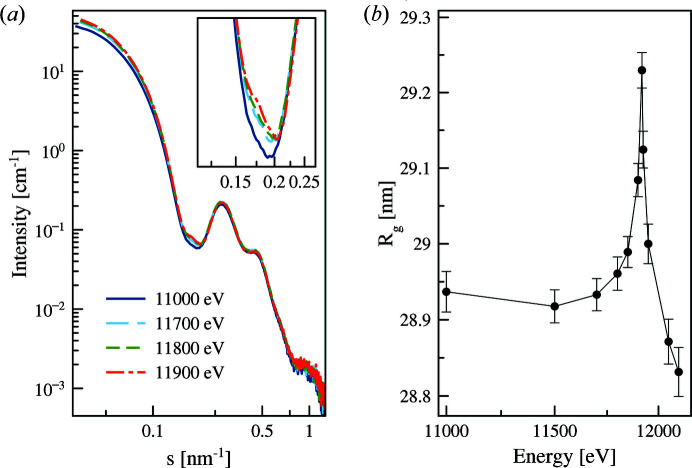
ASAXS from silica-gold nanoparticles. (*a*) Gold core diameter 10 nm: scattering profiles at different energies. The inset shows the zoomed-in area of the same curves plotted in (*a*) emphasizing the range of the maximum variation in the scattering profiles due to the energy change. (*b*) Evolution of the apparent *R*_g_ versus energy (the line is shown as a guide for the eye).

Gold has a strong scattering contrast in comparison with biological macromolecules and can be used for an illustrative ASAXS example of a core-shell sample. Spherical nanoparticles with a gold core and silica shell were measured at 13 energies from 11000 eV to 12000 eV in the vicinity of the *L*_III_ absorption edge of gold (11919 eV). Fig. 9 ▸(*a*) shows the scattering patterns of the nanoparticles at four selected energies below the absorption edge. The anomalous effect in the scattering patterns is noticeable at a relatively large energy offset from absorption edge (500 eV).

Fig. 9 ▸(*b*) displays an increase in the apparent *R*_g_ at the absorption edge confirming that gold atoms are located in the particle core. The *R*_g_ increase (about 0.29 nm) is very small but clearly detectable and consistent with the expected results from the modelling (see Section 4.1).

### 4.3. Gold nanoparticles coated with PEG   

As another test example, we studied gold nanoparticles (AuNPs) covered with polyethyl­ene glycol (PEG) ligands. In contrast to the previous example, the electron density difference of the organic shell to water is 40 e^−^ nm^−3^, even weaker than that for proteins in solution (about 90 e^−^ nm^−3^).

The use of such mixed layers including PEG-based ligands for the functionalization of nanoparticles is a very popular strategy in the context of nanomedicine for improving or enabling various technological or medical applications of nanoparticles. Numerous studies were performed aiming at a detailed understanding of the involved surface chemistry (Kamaly *et al.*, 2012 ▸; Suk *et al.*, 2016 ▸; Jokerst *et al.*, 2011 ▸). The analysis of the organization of the outer PEG layer and PEG layers with introduced molecular modifications by adding ligands to achieve functionality of nanomaterials is therefore of significant practical importance.

It was demonstrated that the thickness of the ligand layer cannot be determined from form factor measurements, because the ligand does not contribute sufficiently to the scattering signal due to its low scattering contrast. It can, however, be obtained by analyzing the SAXS data from concentrated solutions with a sticky hard sphere model, which allows one to indirectly determine the ligand layer size (Schroer *et al.*, 2016 ▸; Schulz *et al.*, 2018 ▸). The study of the shell at diluted conditions is, however, not possible using this approach. Here, ASAXS was used to determine the difference in the non-resonant (sensitive to gold core and PEGMUA ligand) and the resonant (only sensitive to the gold core) terms. For this, nanoparticles with two different PEGMUA lengths (Mw = 2 kDa; 5 kDa) were studied and the results are presented below.

#### 4.3.1. 2 kDa PEGMUA covered gold nanoparticles   

**Figure 10:**
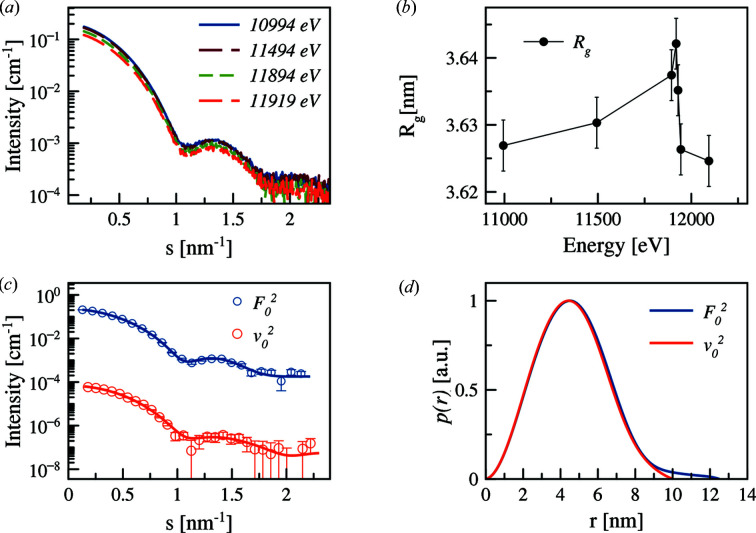
(*a*) Anomalous effect on PEGylated AuNPs with PEG of 2 kDa molecular weight. (*b*) Radius of gyration dependence for energy in the vicinity of the gold absorption edge. An increase of the *R*_g_ indicates that anomalous atoms are concentrated in the core. (*c*) Conventional (blue) and anomalous (red) scattering SAXS curves. Solid lines represent the fits by *GNOM*. The number of shown experimental points is reduced for clarity. (*d*) Normalized pair distance distribution functions from anomalous (red) and conventional (blue) components.

Fig. 10 ▸ displays the SAXS curves of AuNPs coated with PEGMUA (2 kDa) at different X-ray energies close to the gold edge. The presence of characteristic minima of the particle form factor reflects a low dispersity of the gold core (*R* = 4.1 nm; Δ*R*/*R* = 11%). The variations upon energy change are rather weak, but the analysis reveals a small yet systematic increase of *R*_g_ in the vicinity of the absorption edge [Fig. 10 ▸(*b*)]. This finding is consistent with the fact that the anomalous gold atoms are forming a gold core, and, more importantly, that the PEGMUA shell does provide a contribution to the scattering signal (for a pure gold core, no such increase would have been observed).

The analysis was performed using the matrix decomposition method on the collected scattering curves in the vicinity of the absorption edge. The decomposed ASAXS curves are shown in Fig. 10 ▸(*c*) and the computed *p*(*r*) functions do indeed show a difference between the non-resonant and the resonant parts [Fig. 10 ▸(*d*)]. The resonant term, corresponding to the gold core, gives a smaller diameter (*D*_max_ = 10 nm) than the non-resonant term yielding *D*_max_ = 12.5 nm.

#### 4.3.2. 5 kDa PEGMUA covered gold nanoparticles   

**Figure 11:**
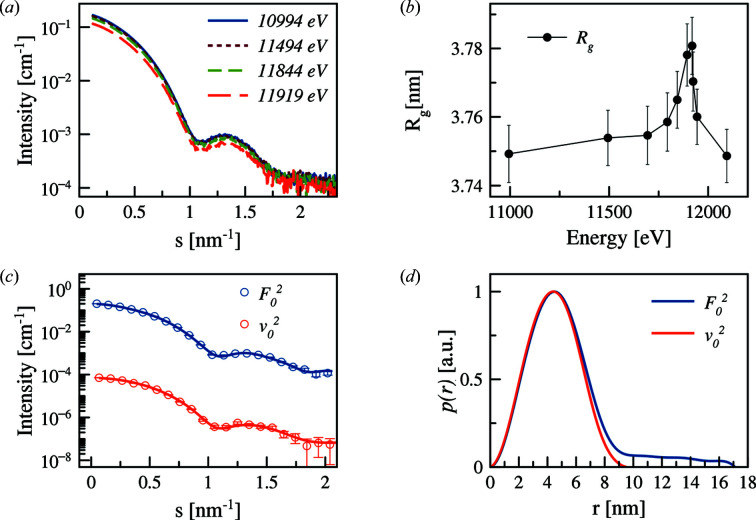
Anomalous effect from PEGylated AuNPs with PEG molecular weight of 5 kDa. The notations are as in Fig. 10 ▸.

Even more pronounced changes are obtained for the 5 kDa PEGMUA shell [Fig. 11 ▸(*a*)]. The *R*_g_ increase is somewhat more significant in the vicinity of the absorption edge [Fig. 11 ▸(*b*)] compared with that observed for 2 kDa. For both samples the *R*_g_ changes are rather small [Δ*R*_g_ (2 kDa) = 0.015 nm and Δ*R*_g_ (5 kDa) = 0.03 nm], but still experimentally detectable.

The decomposed curves for the 5 kDa PEGMUA ligand are displayed in Fig. 11 ▸(*c*). The corresponding pair distance distribution functions *p*(*r*) of the non-resonant and resonant term [Fig. 11 ▸(*d*)] reflect a more pronounced ligand layer compared with 2 kDa PEGMUA, as expected for a longer PEGMUA chain.

**Figure 12:**
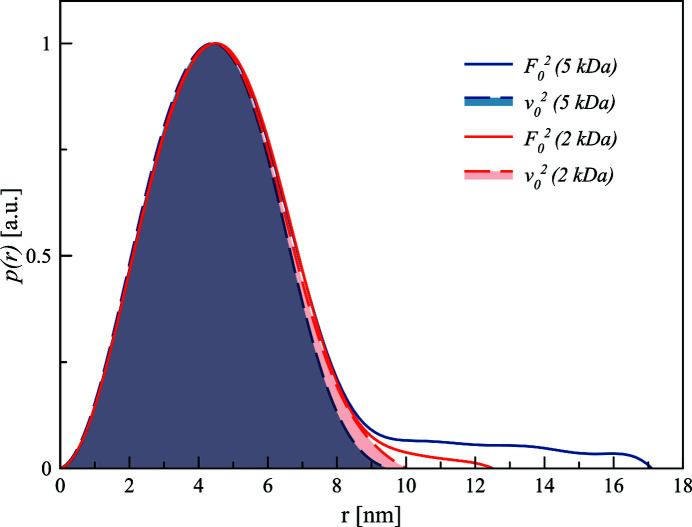
Normalized pair-distance distribution functions *p*(*r*) of anomalous and conventional components of the PEGylated AuNPs. Dotted lines represent the *p*(*r*) for the anomalous part (gold core). Solid lines represent non-anomalous scattering from all atoms in the sample far from the absorption edge. Red is for 2 kDa PEG- and blue for 5 kDa PEG-coated AuNPs. The gold core has the same size in both cases.

The computed pair-distance distribution functions *p*(*r*) for both samples are compared in Fig. 12 ▸. The dotted lines represent the *p*(*r*) for the anomalous part (gold core), the solid lines correspond to the non-anomalous scattering from all atoms in the sample far from the absorption edge (red for 2 kDa PEGMUA and blue for 5 kDa PEGMUA coated gold with approximately the same size of gold core).

The maximum of the *p*(*r*) is at the same position for all curves reflecting the effective gold core radius of 4.5 nm (resulting from the limited size polydispersity). The maximum dimension of the anomalous curves is around 9 nm, which reflects the gold core diameter. The diameters of the AuNPs are 12.5 nm for the 2 kDa PEG sample and 17 nm for the 5 kDa PEG sample.

The overall shapes of the *p*(*r*) functions are rather similar as expected. Differences in the *p*(*r*) are seen in the maximum size of the particle *D*_max_ where *p*(*r*) tends to zero starting from 10 nm for both cases [Figs. 10 ▸(*c*) and 11 ▸(*c*)]. Those differences are less pronounced for 2 kDa PEG-covered nanoparticles and more noticeable for 5 kDa PEG-covered nanoparticles. Therefore, one can check the variation in *D*_max_ using a statistical approach (Section S7). It is shown that variation of *D*_max_ due to the intensity variations of the initial scattering curves and obtained anomalous and non-anomalous contributions is smaller than its absolute values.

Although the PEGMUA ligands are in a dense brush configuration (Schulz *et al.*, 2018 ▸), they do not form a solid shell but the AuNPs’ curvature results in a decreasing PEG segment density protruding away from the gold surface. Thus, the difference in *D*_max_ does not directly correspond to the ligand shell thickness.

Overall, our results demonstrate the possibilities of ASAXS to visualize the contribution of the ligand shell at dilute conditions even for weakly scattering ligands and without assuming any specific structural model. Because the scattering of the ligand shell depends on its density and can be modified, for example, by interactions with cations/salts and/or proteins (Kewalramani *et al.*, 2013 ▸; Spinozzi *et al.*, 2017 ▸), this has potential for the *in situ* characterization of nanoparticle–matrix interactions.

## 5. Conclusion   

The ASAXS technique was established at the P12 BioSAXS beamline (EMBL, Hamburg) and made available in the frame of the user access program. The relevant procedures and options were incorporated into the beamline control software and the data processing pipeline allowing the preliminary results to be rapidly and automatically calculated and immediately provided to the users.

Several model systems including silica-covered gold nanoparticles and micelles with bromine counter ions were studied with ASAXS to verify and demonstrate the capabilities of the setup. The functionalized nanoparticles are shown to be a suitable model system for the anomalous scattering experiments. Using ASAXS, one can determine the structural organization of the outer PEG layer of the particles without assuming an *a priori* model for data interpretation. The ASAXS option is demonstrated to be a useful tool for further characterization of functionalized nanomaterials for medical, pharmaceutical, and biological applications, offering also advanced possibilities to study biological samples.

## 6. Related literature   

The following references, not cited in the main body of the paper, have been cited in the supporting information: Kikhney *et al.* (2020 ▸); Schöps *et al.* (2016 ▸); Tischer *et al.* (2007 ▸); Waldron *et al.* (2009 ▸); Walker (1996 ▸).

## Supplementary Material

Supporting information file. DOI: 10.1107/S1600577521003404/ju5023sup1.pdf
