# Supplementary material for: Anomalous SAXS at P12 beamline EMBL Hamburg: instrumentation and applications
Source: J Synchrotron Radiat. 2021 Apr 14;28(Pt 3):812–23. doi: 10.1107/S1600577521003404 (PMC8127372; doi:10.1107/S1600577521003404)
Supplement: Supplementary file 1 [file s-28-00812-sup1.pdf]

JOURNAL OF SYNCHROTRON RADIATION

Volume 28 (2021)

Supporting information for article:

Anomalous SAXS at P12 beamline EMBL Hamburg: instrumentation and applications

Andrey Yu. Gruzinov, Martin A. Schroer, Karen Manalastas-Cantos, Alexey G. Kikhney, Nelly R. Hajizadeh, Florian Schulz, Daniel Franke, Dmitri I. Svergun and Clement E. Blanchet

S1. Statistics on number of publications

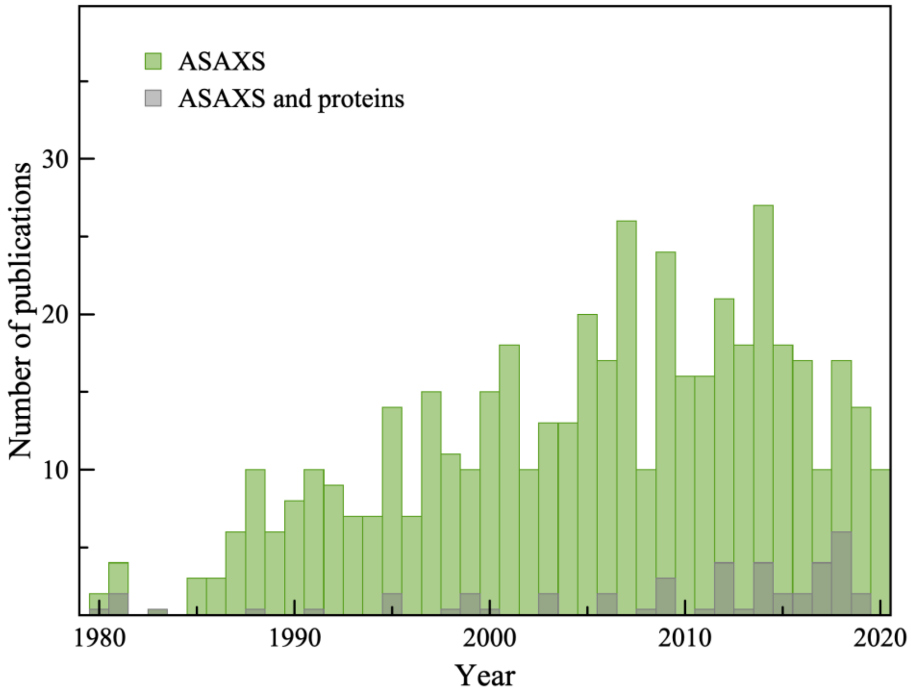

Figure S1 Number of publications mentioning 'ASAXS' and publications mentioning 'ASAXS and proteins' from 1980 to 2020 based on publications search at https://www.dimensions.ai/products/free/.

S2. The elements used as cofactors by enzymes

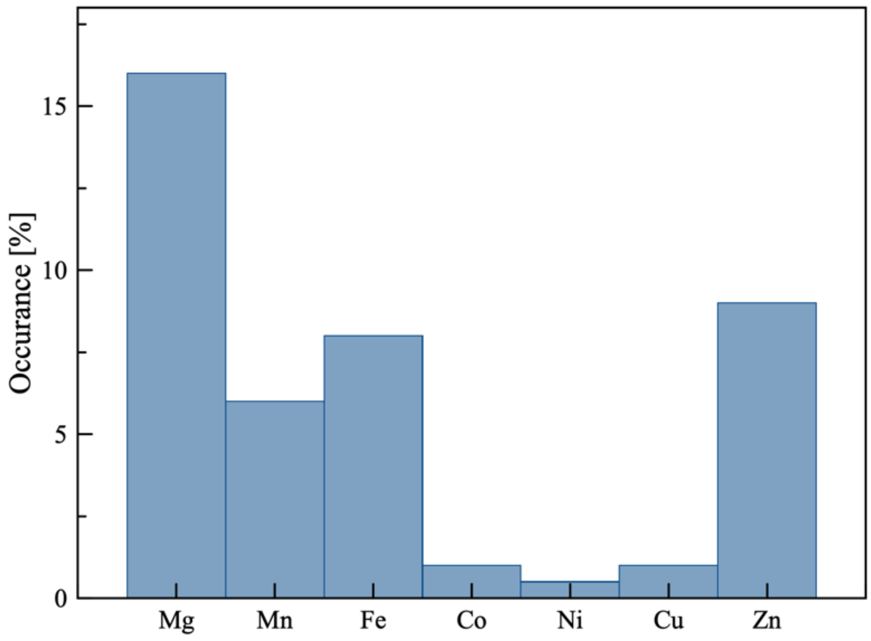

Figure S2 The elements used as cofactors by enzymes. The height of each column represents the proportion of all enzymes with known structures using the respective metal. Adapted from (Waldron et al., 2009).

S3. Energy server calibration and experiment control

S3.1. Energy offset

Beamline interface for the user experiment control is shown in Figure S3. For the energy adjustment, the motor positions are computed for each energy and fine scans around the theoretical positions can be used to optimize the beam flux.

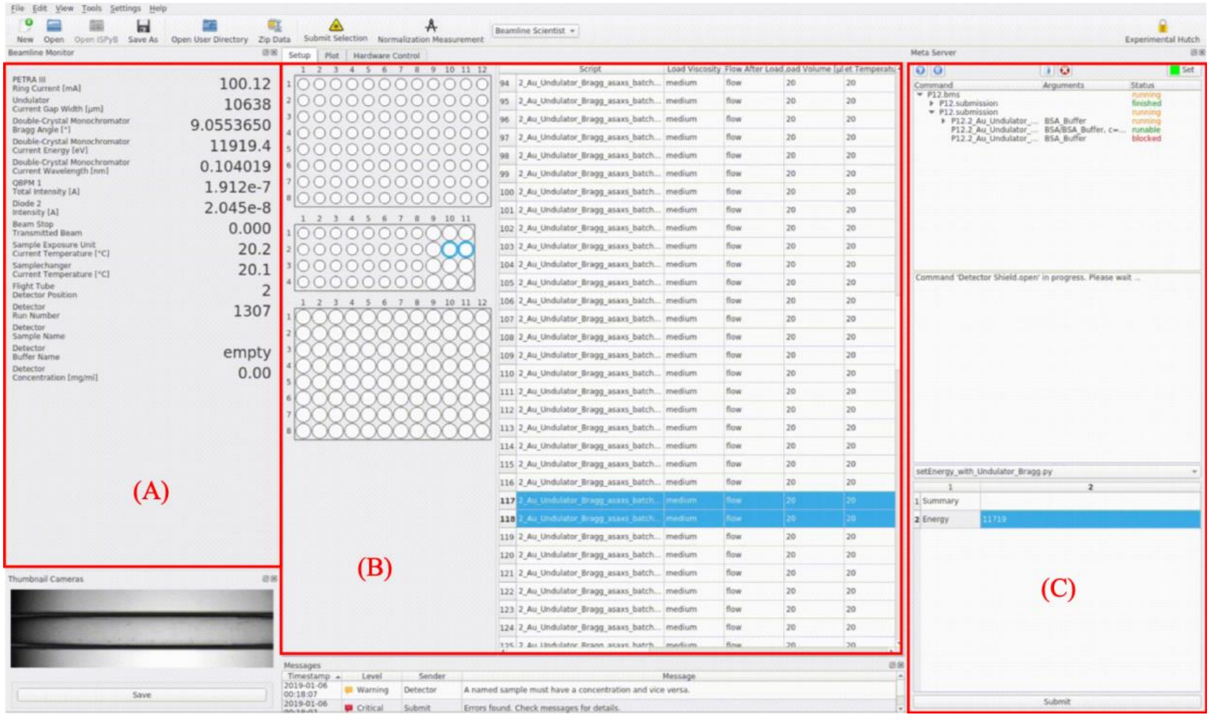

Figure S3 Beamline experiment control interface (BECQUEREL) in ASAXS mode. A) Panel represents beamline parameters and status of the measurement. B) Panel represents sample changer outline with the sample table and loaded scripts for ASAXS data collection. C) Panel shows the queue of measurements and panel with energy setting scripts. Example of the script syntax is listed in SI section S1.4.

To calibrate the energy setting procedure of the double-crystal monochromator (DCM offset), chromium and copper foils are moved into the beam path and their absorption is measured. The measurements of the absorption edges allow to correct for a possible mechanical offset of the Bragg axis of the DCM. Transmission X-ray measurements of solutions with salts containing relevant ions such as iron or bromine, can also be conducted directly in the SAXS cell for fine energy calibration. In addition to the Bragg angle, the undulator gap, the distance between the DCM crystals (perp) and the second crystal alignment (pitch) are adjusted such that the trajectory of the outcoming beam is kept constant for all energies and no further adjustment of the downstream optical elements is required.

S3.2. Undulator gap

Monochromatic X-rays of the P12 BioSAXS beamline are sourced from the controllable-gap low-divergence U29 undulator of PETRA III (Schöps *et al.*, 2016; Tischer *et al.*, 2007). Its known dependence between energy of the main harmonics of undulator and its magnetic properties and storage ring properties is given as:

$$\varepsilon[\mathrm{keV}] = \frac{0.950\mathrm{E}^2[\mathrm{GeV}]\mathrm{n}}{(1+\mathrm{K}^2)\lambda_0[\mathrm{cm}]} \quad \text{(S1)}$$

where K - magnetic parameter, λ_0 - magnetic period of the undulator, E - storage ring energy, n - undulator harmonics (*n*=1 or *n*=3 in for P12). The magnetic parameter reads as:

$$\mathrm{K} = \frac{\mathrm{eB_0}\lambda_0}{2\pi \mathrm{mc}^2} = 0.934\mathrm{B_0}\lambda_0 \quad \text{(S2)}$$

where λ_0 - undulator magnetic period, B_0 - undulator magnetic field, *e* - electron charge, *m* - electron mass, *c* - speed of light.

For PETRA III U29 undulator, it is λ_0 =2.9*cm* and the storage ring energy *E*=6 *GeV.*

The magnetic field B_0 can be empirically estimated as follows (Walker, 1996):

$$\mathrm{B_0} = \mathrm{a}\exp\left(-\mathrm{b}\frac{\mathrm{G}}{\lambda_0} - \mathrm{c}\left(\frac{\mathrm{G}}{\lambda_0}\right)^2\right) \quad \text{(S3)}$$

where *G* - undulator gap, λ_0 - magnetic period of the undulator, *a*, *b*, *c* - empirical parameters for the particular undulator, depending on the material of the magnets and arrangement.

From (S1) we can express the magnetic parameter *K* as follows:

$$\mathrm{K} = \sqrt{\frac{1898\mathrm{E}^2\mathrm{n}}{\epsilon\lambda_0} - 2} \quad \text{(S4)}$$

After substitution (S3) to (S2) and using equation (S4) we obtain following quadratic equation:

$$\frac{\mathrm{c}}{\lambda_0^2}\mathrm{G}^2 + \frac{\mathrm{b}}{\lambda_0}\mathrm{G} - \mathrm{A} = 0 \quad \text{(S5)}$$

where

$$\mathrm{A} = \ln\left(\frac{1}{0.934\mathrm{a}\lambda_0}\sqrt{\frac{1898\mathrm{E}^2\mathrm{n}}{\epsilon\lambda_0} - 2}\right)$$

Solving this equation, one obtains the dependence of the undulator gap G [μm] at the given energy ϵ [keV]:

$$G = \lambda_0 \left(\frac{-b - \sqrt{b^2 + 4Ac}}{2c} \right) \quad \text{(S6)}$$

The second root of the equation (S5) does not fit experimental data. Fitting this equation to the experimental dependence of the undulator gap at different energy settings yield empirical parameters *a*, *b*, *c* of the magnetic structure of the undulator and known harmonic n.

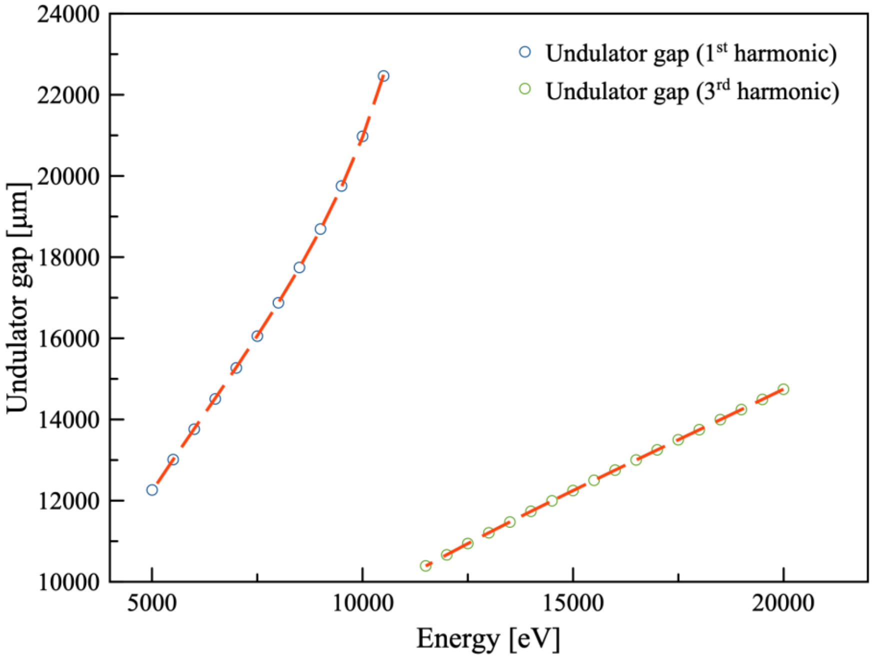

Figure S4 Beamline undulator gap positions measured at different energies (circles) for the 1st and 3rd harmonics. Dashed lines represent fit of equation (S6). Obtained parameters allow one to estimate target undulator gap at any predefined photon energy for beamline tuning.

Figure S4 shows the fitted undulator gap motor position vs energy. The obtained parameters are used for the preliminary setting of the undulator gap prior to the experiment. Monitoring of fitted parameters are available in order to track down changes in gap settings due to the loss of magnetization in undulator.

S3.3. Distance between crystals (Perp)

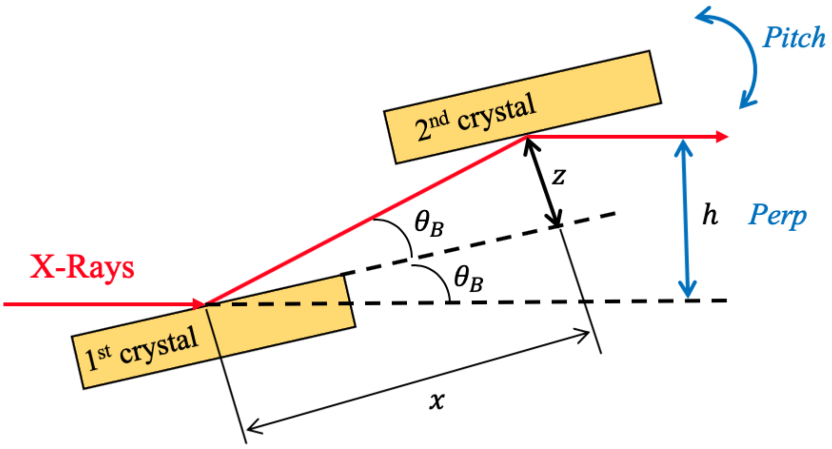

Figure S5 Geometrical scheme of double-crystal monochromator to illustrate the computation of perp.

Vertical distance between incoming and outgoing beams *h* (Figure S5) can be determined using following relation:

$$h = 2z\cos(\theta_B) \quad \text{(S7)}$$

where θ_B - Bragg angle corresponding to the given energy via Bragg's law, *z* - distance between parallel crystal planes. It can be compensated by vertical translation of the crystals in perpendicular direction to each other. Energy selection can be achieved by changing the Bragg angle θ_B which is corresponding to the energy with the well-known Bragg's law. In practical units it can be rewritten as:

$$2d\sin(\theta_B) = \frac{12398}{E[eV]} \quad \text{(S8)}$$

We need to account for the change of the distance between crystals in response to change of the Bragg angle in order to have the beam at the same vertical position. Assume that we slightly changed the energy (Bragg angle) than this corresponds to the following change in vertical distance *h* between the beams:

$$\frac{\delta h}{\delta \theta_B} = -2z\sin\theta_B \quad \text{(S9)}$$

Substitution of *z* from equation (S7) into equation (S9) gives:

$$\frac{\delta h}{h} = -\mathrm{tg}\theta_B \delta\theta_B \quad \text{(S10)}$$

Integration of both parts gives:

$$\ln(h) = \ln\cos\theta_B + const \quad \text{(S11)}$$

or

$$h = h_0 \ln(\cos\theta_B) \tag{S12}$$

Therefore, overall compensation of the vertical change between beam positions can be written as:

$$h = h_0\left(1 + \ln(\cos\theta_B)\right) \tag{S13}$$

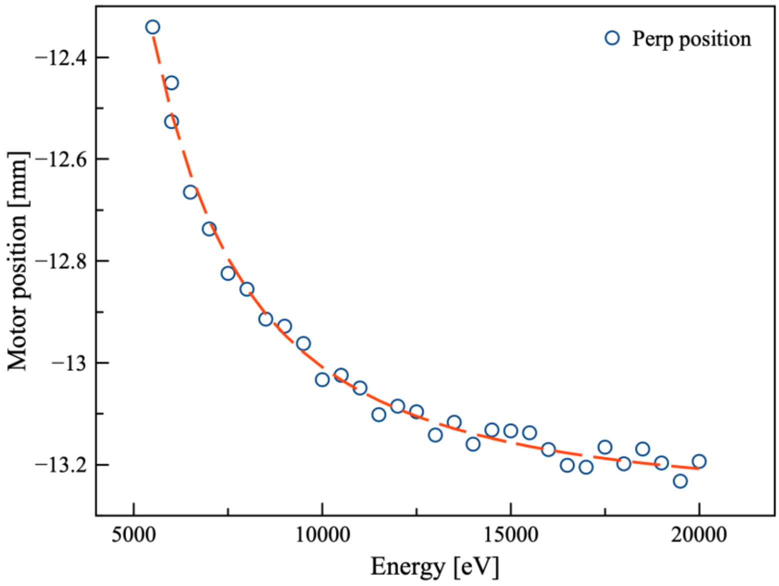

Figure S6 Measured DCM relative perp motor position (circles) at different photon energies. Dashed line represents fit of equation (S13) therefore allowing to calculate optimal perp position of the DCM at an arbitrary energy value.

Equation (S13) was used as a basis for calculation and finding parameters for preliminary distance setting for the DCM. Resulting fit is shown on Figure S6, which emphasizes that correct setting of the perp motor position is more important for the lower energy range than for the higher energy range.

S3.4. Pitch of the second crystal.

Pitch motor of the second crystal is used to ensure the parallelism of two crystal substrates with respect to each other. Slight error in parallelism can cause significant change in the intensity after monochromator. The angle variation should be within the Darwin width of the given set of crystals which is in turn energy-dependent. For preliminary setting of the pitch a cubic polynomial function is used. More precise setting is done via additional scan using piezo-drives with high precision.

S3.5. Example of the script that can be run by user/beamline scientist to perform the calibration of the energy server.

```
from _commands import *
```

```
import PETRAIII.P12.OpticsHutch.DoubleCrystalMonochromator as DCM
import PETRAIII.P12.OpticsHutch.Slit1 as Slit1
import PETRAIII.P12.OpticsHutch.QBPM1 as QBPM1
import PETRAIII.P12.OpticsHutch.QBPM2 as QBPM2
import PETRAIII.P12.ExperimentalHutch.QBPM3 as QBPM3
import PETRAIII.P12.ExperimentalHutch.Cage.BeamStop as BeamStop
import PETRAIII.P12.ExperimentalHutch.BeamConditioningUnit.Attenuator as
Attenuator
import PETRAIII.P12.ExperimentalHutch.BeamConditioningUnit.Diode2 as Diode2
import PETRAIII.P12.ExperimentalHutch.BeamConditioningUnit.Diode1 as Diode1
import
PETRAIII.P12.ExperimentalHutch.BeamConditioningUnit.ExperimentalShutter as
FastShutter
import PETRAIII.P12.OpticsHutch.QBPM1 as QBPM1
import PETRAIII.P12.OpticsHutch.DoubleCrystalMonochromator.Perp as Perp
import PETRAIII.P12.OpticsHutch.DoubleCrystalMonochromator.Pitch as Pitch
import PETRAIII.P12.FrontEnd.Undulator as Undulator



import numpy as np
import time
import datetime



def Bragg_from_Energy_Si111(energy):
    En_lambda_const = 6.62606896e-34*299792458/1.60217687e-19*1
    return
round(np.arcsin(En_lambda_const/(energy*2.0*3.1356))*180.0/np.pi,7)


def getPerp_from_Energy(energy):

    p0=-13.264554890940
    p=p0*(1.0+np.log(np.cos(Bragg_from_Energy_Si111(energy)*np.pi/180)))

    return p


def Energy_from_Bragg_Si111(bragg):
```

```
    En_lambda_const = 6.62606896e-34*299792458/1.60217687e-19*1
    energy = En_lambda_const/(2.0*3.1356*np.sin((bragg)*np.pi/180.0)) #eV

    return energy


def getUndulatorGap_from_Energy(energy):

    energy = energy/1000.0 #input energy in eV
    En_switch = 11  # Switch between the harmonics (energy, in keV)

    E_ring = 6 #Ring energy in GeV
    lamb = 2.9 #Undulator period in cm


    if energy<En_switch:

        #1st harmonics

        n_harm = 1 # harmonic number
        a=2.1514765
        b=-0.2781146
        c=-0.00504


A=np.log(1.0/(a*0.934*lamb)*np.sqrt((1.898*E_ring**2*n_harm)/(energy*lamb)-
2.0))

        Gap_1st_harm =  lamb*((-b-np.sqrt(b**2+4.0*A*c))/(2*c))

        return Gap_1st_harm*1000 #in mm


    if energy>=En_switch:

        #3rd harmonics

        n_harm = 3 # harmonic number
        a=2.515237
        b=-0.34108303
```

```
        c=-0.0001158347


A=np.log(1.0/(a*0.934*lamb)*np.sqrt((1.898*E_ring**2*n_harm)/(energy*lamb)-
2.0))

        Gap_1st_harm =  lamb*((-b-np.sqrt(b**2+4.0*A*c))/(2*c))

        return Gap_1st_harm*1000 #in mm


def getPitch_from_polynomial_fit(energy):

    a0 = -0.451677833328
    a1 = 1.49883864339e-06
    a2 = -5.24596874534e-11

    pitch = a0+a1*energy+a2*energy**2

    return pitch


def SetEnergy_P12_with_fine_scan(energy,MONITOR,**kwargs):

    dt = datetime.datetime.now().strftime("%Y-%m-%d_%H-%M-%S")

    AngularBraggOffset = -0.4881232077
    BraggTheory = Bragg_from_Energy_Si111(energy)
    BraggMotorPosition = BraggTheory+AngularBraggOffset


    theory_gap = round(getUndulatorGap_from_Energy(energy),0)
    theory_perp = getPerp_from_Energy(energy)
    theory_pitch_from_polynomial_fit = getPitch_from_polynomial_fit(energy)


    DCM.setBraggAngle(BraggMotorPosition)
    Undulator.setGapWidth(theory_gap)
    Pitch.setVelocity(0.1)
    Pitch.moveAbsolute(theory_pitch_from_polynomial_fit)
    Perp.setVelocity(0.1)
```

```
    Perp.moveAbsolute(theory_perp)



    #Fine undulator scan
    #====================

    prefix = kwargs["Log Directory"] +
"/"+dt+"_Undulator_Gap_vs_QBPM1_QBPM2_QBPM3_Diode2_EnergyServer_"+str(energ
y)
    Undulator.scanGapWidth(theory_gap-300,theory_gap+600,25,MONITOR,prefix)
    Undulator.fitPdf(prefix)
    Undulator.setGapWidthPercentile(prefix,0.500)

    #Fine perp scan
    #===============

    Perp.setVelocity(0.1)
    prefix = kwargs["Log Directory"] +
"/"+dt+"_Perp_vs_QBPM1_QBPM2_QBPM3_Diode2_EnergyServer_"+str(energy)
    Perp.scanAbsolutePosition(theory_perp-
1.0,theory_perp+1.0,0.05,MONITOR,prefix)
    Perp.fitPdf(prefix)
    Perp.setAbsolutePositionPercentile(prefix,0.500)



    #Fine pitch scan
    #===============

    Pitch.setVelocity(0.1)
    prefix = kwargs["Log Directory"] +
"/"+dt+"_Pitch_vs_QBPM1_QBPM2_QBPM3_Diode2_EnergyServer_"+str(energy)
    Pitch.scanAbsolutePosition(theory_pitch_from_polynomial_fit-
0.05,theory_pitch_from_polynomial_fit+0.05,0.001,MONITOR,prefix)
    Pitch.fitPdf(prefix)
    Pitch.setAbsolutePositionPercentile(prefix,0.500)
```

```
def experiment(*args, **kwargs):

# ================ Description ===================================
# This script goes through "Energy_list" and do a fine scans
# of undulator gap, perp and pitch around approximate positions.
# ================================================================

# Chose a list of intensities to be written into the file.

    MONITOR =
[QBPM1.TotalIntensity,QBPM2.TotalIntensity,QBPM3.TotalIntensity,Diode2.Inte
nsity]
    # In eV
    Energy_list =
[6000,6500,7000,7500,8000,8500,9000,9500,10000,10500,11000,11500,12000,1250
0,13000,13500,14000,14500,15000,15500,16000,16500,17000,17500,18000,18500,1
9000,19500,20000]


    # Reference energies
    #Cr_Edge = 5989
    #Energy_list = [Cr_Edge]

    #Cu_Edge = 8979

    #Energy_list = [Cu_Edge]

    for energy in Energy_list:
        SetEnergy_P12_with_fine_scan(energy,MONITOR,**kwargs)

    SetEnergy_P12_with_fine_scan(10000,MONITOR,**kwargs)
```

S4. List of elements and absorption edges

Table 1 Elements and their corresponding absorption edges (in eV) from 3500 to 20 000 eV Adapted from https://xdb.lbl.gov.

Element	K		Element	LI	LII	LIII		Element	LI	LII	LIII
K	3608.4		Pd	3806	3524			Lu	10890	10349	9244
Ca	4038.5		Ag	3806	4238			Hf	11271	10739	9561
Sc	4492		Cd	4018	3727	3538		Ta	11682	11136	9881
Ti	4966		In	4238	3938	3730		W	12100	11544	10207
V	5465		Sn	4465	4156	3929		Re	12527	11959	10535
Cr	5989		Sb	4698	4380	4132		Os	12968	12385	10871
Mn	6539		Te	4939	4612	4341		Ir	13419	12824	11215
Fe	7112		I	5188	4852	4557		Pt	13880	13273	11564
Co	7709		Xe	5453	5107	4786		Au	14353	13734	11919
Ni	8333		Cs	5714	5359	5012		Hg	14839	14209	12284
Cu	8979		Ba	5989	5624	5247		Tl	15347	14698	12658
Zn	9659		La	6266	5891	5483		Pb	15861	15200	13035
Ga	10367		Ce	6549	6164	5723		Bi	16388	15711	13419
Ge	11103		Pr	6835	6440	5964		Po	16939	16244	13814
As	11867		Nd	7126	6722	6208		At	17493	16785	14214
Se	12658		Pm	7428	7013	6459		Rn	18049	17337	14619
Br	13474		Sm	7737	7312	6716		Fr	18639	17907	15031
Kr	14326		Eu	8052	7617	6977		Ra	19237	18484	15444
Rb	15200		Gd	8376	7930	7243		Ac	19840	19083	15871
Sr	16105		Tb	8708	8252	7514					
Y	17038		Dy	9046	8581	7790					
Zr	17998		Ho	9394	8918	8071					
Nb	18986		Er	9751	9264	8358					
Mo	20000		Tm	10116	9617	8648					
Tc	21044		Yb	10486	9978	8944					

S5. Estimation of constant fluorescence background from ASAXS data

The fluorescent signal is estimated by monitoring the change in the background in the SAXS data. Automatic estimation of the fluorescence background was built on method of over-subtraction determination for macromolecular SAXS data. Over-subtracted data usually produced by a mismatch between buffer used for sample preparation and the pure buffer measurement that is used later for the automated subtraction procedure. Detection of over-subtracted data is implemented as a component of the automated SAXS data analysis pipeline SASFLOW (Franke *et al.*, 2012) and in the curated repository for small angle scattering data and models SASBDB (Kikhney *et al.*, 2020).

Let us define "over-subtraction" as the presence of one or more systematically negative data ranges in the background-subtracted scattering curve. For any given over-subtracted scattering curve there is a minimum constant that can be added to the data to make it not over- subtracted. This constant is found using a binary search. of the Longest Consecutive Negative Sequence (LCNS) of intensity values. As a criterion for detection of such systematically negative intensity subsets is likely to occur by chance we have adopted the Correlation Map (Franke *et al.*, 2015). The search for LCNS is repeated multiple times on the same data after averaging every two, three, four etc. subsequent points until either the data is identified as over-subtracted or a predefined minimal number of averaged data points in the scattering curve is reached. In the latter case the curve is considered not over- subtracted.

The problem of detection of "fluorescence" constant from the one-dimensional data curve can be treated as problem of "under-subtraction" when the buffer-subtracted curves still show a significant constant offset. "Under-subtraction" is treated as an inverse oversubtraction problem. The over-subtraction detection approach was adapted to find a maximum constant that can be subtracted from the experimental data without making it systematically negative.

The constant determined by this method reflects the contribution of both scattering of the solute and of the fluorescence. Assuming that the anomalous signal is weak, especially in the region where the scattering intensity are low, the change in the constant at different energies can be largely attributed to the change in the fluorescent signal. Comparison between the determined constants far below the absorption edge and around the absorption edge provides an estimation of fluorescence signal contribution to the processed data.

Therefore, this constant is only used as an approximate estimation of the fluorescence signal and should be used with caution. Generally speaking, the determined constant value is the sum of fluorescence, angle independent fluctuation scattering and resonant Raman scattering. It allows a rapid assessment of the quality of fluorescence corrected data and can be further adjusted if needed.

S6. Statistics on the PDB entries with corresponding elements

Table 2 Statistics on PDB entries containing elements. Ratio shows percentage of structures available in the PDB that contain the element of interest (as of April 2020, https://www.rcsb.org).

Element	Number of entries	Ratio of total entries, %
Zn	16125	9,94
Ca	11465	7,07
Se	9715	5,99
Fe	9226	5,69
Mn	3632	2,24
K	2901	1,79
Ni	2010	1,24
Br	1894	1,17
Cu	1728	1,06
I	1390	0,86
Co	1284	0,79
Cd	1038	0,64
As	702	0,43
Hg	675	0,42
Pt	271	0,17
Mo	248	0,15
Ba	184	0,11
Sr	165	0,1
V	163	0,1
Xe	137	0,08
Cs	131	0,08
W	122	0,08
Au	107	0,07
Yb	92	0,06

Gd	81	0,05
Pb	65	0,04
Ir	64	0,04
Rb	63	0,04
Y	62	0,04
Os	61	0,04
Tl	56	0,03
U	54	0,03
Sm	48	0,03
Ag	40	0,02
Pr	38	0,02
Tb	37	0,02
Pd	27	0,02
Re	27	0,02
Eu	24	0,01
Kr	21	0,01
Ta	21	0,01
Te	16	0,01
Lu	15	0,01
La	14	0,01
Cr	12	0,01
Ga	12	0,01
Ho	12	0,01
Sn	9	0,01

S7. Modelling of D_{max} variation for PEGMUA covered gold nanoparticles.

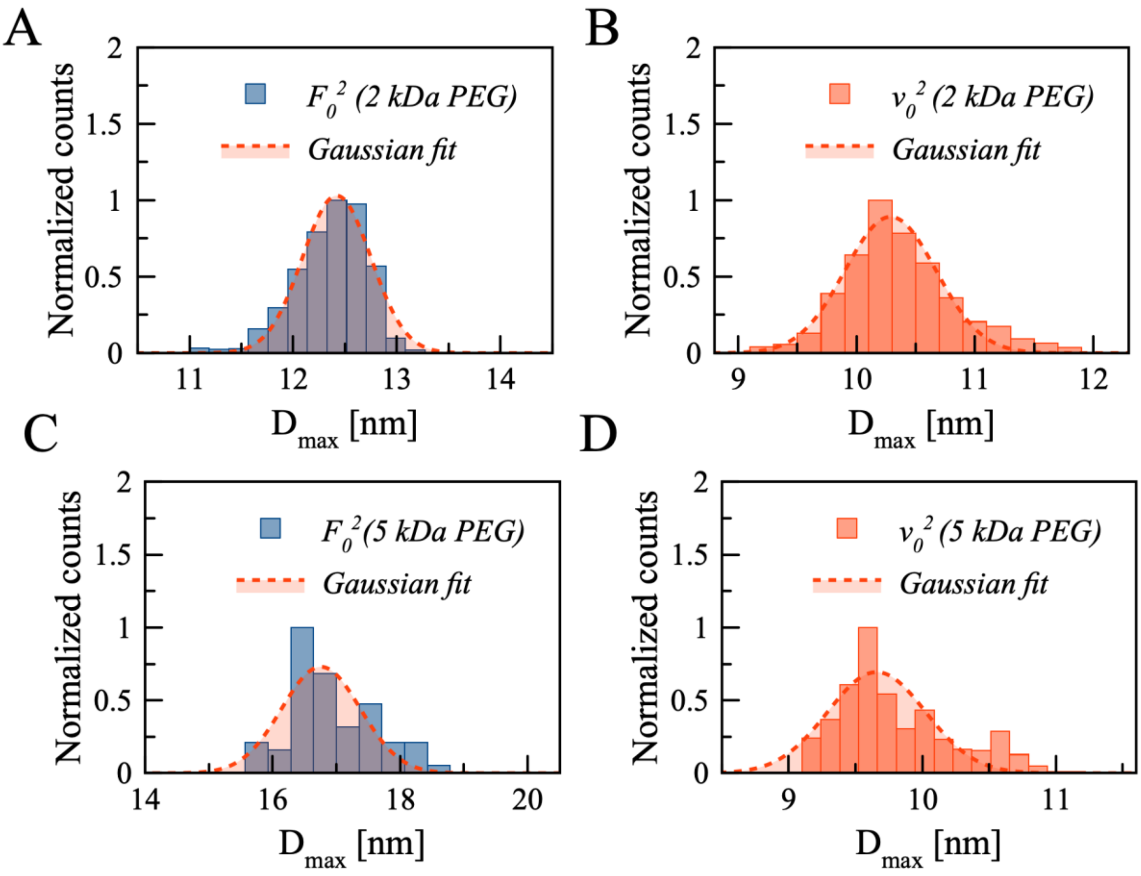

Figure S7 Normalized D_{max} distributions for anomalous and non-anomalous components. A) $F^2{}_0$ 2 kDa PEGMUA coated gold nanoparticles. B) v_0^2 2 kDa PEGMUA coated gold nanoparticles. C) $F^2{}_0$ 5 kDa PEGMUA coated goldv nanoparticles. D) v_0^2 5 kDa PEGMUA coated gold nanoparticles.

In order to estimate the difference in D_{max} in resulting $p(r)$ functions, we have computed $p(r)$ functions for the partial intensities F_0^2 and v_0^2 obtained by matrix decomposition of the 1000 ASAXS datasets generated from original dataset. Generation was done by small variations of the intensities of the original curves within the experimental errors using DATRESAMPLE software from ATSAS package (Manalastas et al., 2020). Resulting distributions of D_{max} values are shown on the Figure S7.

D_{max} values are 10.3+-0.5 nm for anomalous component and 12.4+-0.4 nm for 2 kDa PEG coated nanoparticles; 9.6+-0.4 and 16.8+-0.7 nm for 5 kDa PEG coated nanoparticles respectively. Those values are in a good agreement with the values obtained from a single $p(r)$ (10 and 12.5; and 9.5 and 17 nm respectively). As it can be seen the differences in p(r) between the F_0^2 and v_0^2 are indeed small but statistically significant and can be used as an estimate for the sizes of the core and shells of the particles.
